# IntPath--an integrated pathway gene relationship database for model organisms and important pathogens

**DOI:** 10.1186/1752-0509-6-S2-S2

**Published:** 2012-12-12

**Authors:** Hufeng Zhou, Jingjing Jin, Haojun Zhang, Bo Yi, Michal Wozniak, Limsoon Wong

**Affiliations:** 1 NUS Graduate School for Integrative Sciences & Engineering, National University of Singapore, Singapore; 2 School of Computing, National University of Singapore, Singapore; 3 Faculty of Mathematics, Informatics and Mechanics, University of Warsaw, Poland

## Abstract

**Background:**

Pathway data are important for understanding the relationship between genes, proteins and many other molecules in living organisms. Pathway gene relationships are crucial information for guidance, prediction, reference and assessment in biochemistry, computational biology, and medicine. Many well-established databases--e.g., KEGG, WikiPathways, and BioCyc--are dedicated to collecting pathway data for public access. However, the effectiveness of these databases is hindered by issues such as incompatible data formats, inconsistent molecular representations, inconsistent molecular relationship representations, inconsistent referrals to pathway names, and incomprehensive data from different databases.

**Results:**

In this paper, we overcome these issues through extraction, normalization and integration of pathway data from several major public databases (KEGG, WikiPathways, BioCyc, etc). We build a database that not only hosts our integrated pathway gene relationship data for public access but also maintains the necessary updates in the long run. This public repository is named IntPath (**Int**egrated **Path**way gene relationship database for model organisms and important pathogens). Four organisms--*S. cerevisiae, M. tuberculosis *H37Rv, *H. Sapiens *and *M. musculus*--are included in this version (V2.0) of IntPath. IntPath uses the "full unification" approach to ensure no deletion and no introduced noise in this process. Therefore, IntPath contains much richer pathway-gene and pathway-gene pair relationships and much larger number of non-redundant genes and gene pairs than any of the single-source databases. The gene relationships of each gene (measured by average node degree) per pathway are significantly richer. The gene relationships in each pathway (measured by average number of gene pairs per pathway) are also considerably richer in the integrated pathways. Moderate manual curation are involved to get rid of errors and noises from source data (e.g., the gene ID errors in WikiPathways and relationship errors in KEGG). We turn complicated and incompatible xml data formats and inconsistent gene and gene relationship representations from different source databases into normalized and unified pathway-gene and pathway-gene pair relationships neatly recorded in simple tab-delimited text format and MySQL tables, which facilitates convenient automatic computation and large-scale referencing in many related studies. IntPath data can be downloaded in text format or MySQL dump. IntPath data can also be retrieved and analyzed conveniently through web service by local programs or through web interface by mouse clicks. Several useful analysis tools are also provided in IntPath.

**Conclusions:**

We have overcome in IntPath the issues of compatibility, consistency, and comprehensiveness that often hamper effective use of pathway databases. We have included four organisms in the current release of IntPath. Our methodology and programs described in this work can be easily applied to other organisms; and we will include more model organisms and important pathogens in future releases of IntPath. IntPath maintains regular updates and is freely available at http://compbio.ddns.comp.nus.edu.sg:8080/IntPath.

## Background

The proliferation of pathway databases--e.g., KEGG [1], WikiPathways [2,3], BioCyc [4,5], and MouseCyc [6]--are useful for understanding the relationship between genes, proteins and other molecules in living organisms. However, the effectiveness of these databases is hindered by issues such as incompatible data formats, inconsistent molecular representations, inconsistent molecular relationship representations, inconsistent referrals to pathway names, and incomprehensive data from different databases. These difficulties call for an effective integration of these databases.

There are many approaches to integrate pathways. For example, Pathway Commons and PathCase [7] can be considered as taking the "aggregator" approach. In this approach, a common access method and data format are adopted or developed for a set of pathways imported from a collection of source databases. The aggregator approach does not perform any unification of the underlying pathways--viz., if *n *source databases each contains information on a particular pathway, that pathway is presented by the aggregator as *n *separate pathways. On the other hand, GenMapp [8], Cytoscape [9], and PathVisio [10] can be considered as taking the "converter" approach. Basically, these tools support the import and export of biological pathways in a variety of formats, even though these tools are designed mainly for exploring, visualizing, and editing biological pathways. Lastly, PathwayAPI [11] can be considered as taking the "full unification" approach. In this approach, pathways in different source databases that are meant to represent the same pathway are merged and molecular objects mentioned in the different source pathways that are meant to represent the same objects are matched. This approach is technically more difficult than other approaches; but it has the advantage of presenting a more coherent and comprehensive view of the pathways.

Very recently, Stobbe et al. [12] compared the genes, EC numbers and reactions of five frequently used human metabolic pathway databases. They found that the overlap between these databases is surprisingly low. More importantly, their results show that each of the five networks compared provides a valuable piece of the puzzle of the comprehensive reconstruction of the human metabolic network. This discovery is a strong motivation for the "full unification" approach mentioned above. Stobbe et al. further suggested that, for an effective integration, one needs to standardize the metabolite names and identifiers and to resolve the conceptual differences between the databases.

Besides the databases that focus specifically on pathway data integration, some protein functional interaction databases have also extended their collection to pathway data. For example, ConsensusPathDB [13] integrates different types of functional interactions from heterogeneous interaction data resources and pathway databases for three organisms(human, yeast and mouse).The distinct difference in their primary focus results in an obvious difference between ConsensusPathDB and IntPath. ConsensusPathDB collects pathway data from many databases but dose not appear to produce integrated pathways--even when the same pathway is present in different sources, they are still listed individually without merging. How to merge the different instances of the same pathways among and within the source pathway databases is the major concern of IntPath. Unlike ConsensusPathDB, IntPath mainly focuses on the integration of pathway-gene and pathway-gene pair relationships, with the aim of solving the problem of inconsistencies and incomprehensiveness among different pathway databases. The definition of "gene pair" in this paper is the gene-gene relationship in pathways, the relationship type of the two components in each gene pair is described in table 1.

**Table 1:** Four types of IntPath unified gene relationships.

Unified Genes Relationships	Explanation
ECrel	Enzyme-enzyme relation, indicating two enzymes catalyzing successive reaction steps.
PPrel	Protein-protein interaction, such as binding and modification, or proteins have control over the same process.
GErel	Gene expression interaction, indicating relation of transcription factor and target gene product.
GPrel	Proteins belong to the same molecular complex, not necessarily interacting directly.

Explanations of the types of relationships in IntPath are given below.

In this paper, we take this full unification approach in building **Int**Path, the **Int**egrated **Path**way gene relationship database for model organisms and important pathogens. This approach was also taken earlier by Soh et al. [11] when they integrated general human pathways into PathwayAPI. IntPath differs from PahtwayAPI in several aspects. In terms of content, a different set of databases and multiple organisms are considered in IntPath. In terms of data extraction, IntPath extracts all pathway data directly from the xml files of each source database and the whole process is highly automated. Therefore, IntPath provides integrated and unified pathway information on a much larger set of organisms and it can be extended to include many other organisms in a short time. In contrast, PathwayAPI integrated only human pathways. Also, for all the organisms included in IntPath, a regular update of each organism can be maintained. In terms of pathway data integration, IntPath not only looks for related pathways between databases but also within each source database; this integration approach provides more unified, meaningful and comprehensive integrated pathway-gene and pathway-gene pair relationships information. In contrast, PathwayAPI only looks for related pathways between databases but not within the same source database. Moreover, IntPath also provides more features and tools. It not only supports web service but also a full-featured web interface. More analysis tools based on pathway data have been provided--like "Analyze Distance" and "Identify Pathways"--and more analysis functions and tools will continue to be added on IntPath in future releases.

The incompatible data formats of different databases seriously inhibit effective and compatible information retrieval. In KEGG, pathways are represented in KGML format and SOAP (returned when using API calls). In WikiPathways, pathways are represented in GPML format; recently it begins to support web service [3], allowing users to access the data through API calls; and the BioPAX format is also supported. In BioCyc and MouseCyc, the pathway data are primarily represented in the BioPAX format. In IntPath, we overcome this limitation by extracting the pathway gene relationships from these different databases and convert these various complicated XML-based formats into simple tab-delimited text files.

Inconsistent molecular representations significantly lower the effectiveness of pathway information retrieval. Different databases maintain different naming conventions on the nodes of their pathways. In KEGG, the names of the nodes (genes and proteins) in the pathways can be KEGG Entry name, KEGG ORTHOLOG (KO) ID, etc. The graphic names on KEGG pathway map can be Gene Symbol (or synonym), Enzyme Commission (EC) number, etc. In WikiPathways, the nodes' "TextLabel" are given gene symbol (or synonym), gene name, protein name, EC number, etc. In most cases the nodes can also be given Entrez ID, NCBI Accession, Ensembl Gene ID, Ensembl protein ID, UniProt Assession, etc. And some times nodes in WikiPathways are only given "TextLabel" without any database reference ID. MouseCyc [6] mainly uses MGI ID and also includes the corresponding gene symbol, UniProt accession, etc. BioCyc (MTBRvCyc, YeastCyc and HumanCyc) Accession Number is mainly used to represent nodes in the pathways while corresponding gene symbol, gene name (protein name), Entrez ID, and UniProt accession number are sometimes included. Inconsistent molecular relationship representations may also cause confusion when referencing pathway information from different repositories. In KGML (KEGG), the relationships between molecules are represented as PPrel, ECrel, PCrel, GErel, etc. In GPML (WikiPathways), the relationships can be inhibition, activation, protein complexes, enzyme complexes, acetylation, phosphorylation, etc. In BioPAX (BioCyc and MouseCyc), when transformed into the SIF format, the relationships can be SEQUENTIAL_CATALYSIS, CO_CONTROL, INTERACTS_WITH, IN_SAME_COMPONT, METABOLIC_CATALYSIS, etc. These inconsistencies cause troubles for researchers wishing to refer to pathway information in a large-scale manner across different databases. Therefore, some normalization technique is needed to convert the nodes and edges from different pathways in different repositories into a common representation. In IntPath, we overcome the above two limitations by normalizing the pathway gene representations and gene relationship representations from different databases into unified IntPath gene and relationship representation. The unified IntPath gene ID for *Homo Sapiens *is HGNC Symbol, *Mus musculus *is MGI Symbol, *Saccharomyces cerevisiae *is Systematic name, and *Mycobacterium tuberculosis *H37Rv is TuberList Rv number. The unified IntPath gene relationship representations are listed in Table 1.

Inconsistent referrals to pathway names are another source of confusion that substantially reduces the effectiveness of retrieving information on the same pathway from different databases. For instance, KEGG may refer to a pathway as "Glycolysis/"Gluconeogenesis", and WikiPathways may name it as "Glycolysis and Gluconeogenesis". For another example, WikiPathways contains a pathway with the name "Cholesterol Biosynthesis", while BioCyc has many corresponding pathways such as "cholesterol biosynthesis III (via desmosterol)", "cholesterol biosynthesis II (via 24, 25-dihydrolanosterol)", "cholesterol biosynthesis I", and "superpathway of cholesterol biosynthesis". Therefore, a unified pathway naming system may reduce the confusion when referring to the same or similar pathway information from different databases.

Furthermore, the comprehensiveness of data from different databases is another limitation of these pathway databases. By the term "incomprehensiveness", we mean that each single biological database is not a comprehensive representation of biological knowledge that is considered by experts to be accurate [11]. We reveal the incomprehensiveness of current databases via analysis on the agreement of the common pathway between these different databases. In IntPath, these inconsistencies and incomprehensiveness issues are solved by the integration approach.

### Data

We choose several representative data sources--KEGG [1], WikiPathways [2,3], BioCyc [4,5], and MouseCyc [6]--for our analysis and integration. These data sources are selected because they are representatives of very different kinds of curation efforts. Currently, the following organisms are included in our IntPath database (version 2.0): *Homo sapiens*, *Mus musculus*, *Saccharomyces cerevisiae *and *Mycobacterium tuberculosis *H37Rv. For each organism included in IntPath, the pathway data are collected from three representative databases: 1. KEGG; 2. WikiPathways; 3. One of the following four databases--YeastCyc, HumanCyc [14], and MTBRvCyc from the BioCyc collection [4,5]; and MouseCyc [6]. The four Pathway/Genome Databases (PGDBs)--MouseCyc, YeastCyc, HumanCyc, MTBRvCyc--are generated and recorded in a very similar way, but the PGDBs of different organisms are maintained and curated by different groups.

MouseCyc is curated by the Jackson Laboratory; it is a new, manually curated database of both known and predicted metabolic pathways for the laboratory mouse [6]. YeastCyc is a Tier-1 PGDB from the BioCyc collection [4,5]; it is curated by SGD Curators in Stanford University. PGDBs in Tier 1 have received more than one year of literature-based curation by scientists. MTBRvCyc and HumanCyc [14] are Tier-2 PGDBs from the BioCyc collection; they are generated by the PathoLogic program and received moderate curation (mostly have undergone 1-4 months of curation). WikiPathways is maintained by a community of users via a wiki-style platform [2,3]. KEGG database is curated independently by a single lab from published literature [11].

## Methods

### Extraction and normalization of pathway-gene and pathway-gene pair relationships

The first step of extracting information from pathway databases is downloading the XML files. To automatically download the hundreds of KGML files of each organism on the KEGG ftp site, we use a simple spider program written in Perl. For BioCyc and MouseCyc, the BioPAX files--and for WikiPathways, the GPML files--are compressed into a single package which can easily be downloaded manually.

Extracting the pathway-gene and pathway-gene pair relationships from KGML is accomplished using an in-house Java program, which extensively uses regular expressions to retrieve specific information from the KGML files. A KGML file consists of entries like "</entry>", "</relation>" and "</reaction>"; in each entry there is either entry information of the nodes (genes, enzymes, compounds, ortholog groups and so on), groups (complexes of gene products like protein complexes and so on) or relationships (relationship between the nodes in the pathway map). Using regular expressions we can specifically obtain the genes of each pathway and the relationships between each gene, and then link these genes according to the relationships. For genes belonging to complexes (groups), the binary gene pairs are generated based on the matrix model.

An alternative way of retrieving KEGG pathway genes and gene pairs is by calling the KEGG API, which enables users to easily use their programs to get access to the KEGG database. However, the API is not well updated [11]. The KEGG API does provide a function that can retrieve gene relationships from this database, though the results returned are KGML entry IDs, not exactly as we have wanted. Although calling the KEGG API would work in theory as described in [11], we turn to mining KGML directly and achieve the same good results.

Extracting pathway-gene and pathway-gene pair relationships from a GPML file is also accomplished using a strategy similar to mining KGML files. Mining a GPML file is much more difficult due to its wikistyle; and there are slight variations among individual GPML files even in the same organism, like some key tags may be in upper case in one file but lower case in another, random insertions of whitespace character, etc. Due to these variations, the regular expressions used for performing the extraction must be very robust. In GPML files, the information of genes and proteins are stored in a "</DataNode>" entry where, if the node is a gene or protein, the Type of the entry is set to "GeneProduct". The information of relationships (like activation and inhibition) are stored in a "</Line>" entry. The linkage of "</DataNode>" entry and "</Line>" entry is mainly accomplished by their "graphID". A "</Line>" entry usually records which two "</DataNode>" entries it links to through the records of two corresponding "graphID" of the "</DataNode>" entries. Using this information we can retrieve the relationship of two genes linked by a "</Line>" entry. This relationship--like inhibition, activation, etc.--can be regarded as equivalent to the "PPrel" relationship in KGML.

If the genes belong to certain complexes (groups), their "GroupRef" ID are recorded in some "</DataNode>" entries; and genes with same "GroupRef" ID are in the same group (molecular complexes). All possible pair-wise relationships among members of a group are generated based on the matrix model. These relationships, derived from such a group, are mainly binary relationships among members in a protein complex or enzyme complex. They can thus be regarded as equivalent to the "group" relationship in KGML. This strategy works well for most GPML files; but, for some individual files, there is simply no "graphID" in the file and, only positional information of each entry is recorded. This causes difficulty in retrieving the corresponding gene relationships. Attempts have been made to retrieve the pair-wise relationships based purely on the positional information; but these attempts also introduce a substantial amount of noise. Therefore we do not use this noisy information.

Retrieving gene relationships from BioPAX files is mainly by using the Paxtools Java programming library [15] in combination with our own simple Java program. By transforming a BioPAX (Level 2) file into the SIF format, we get both a Node file and an Edge file. Then a simple node mapping is made to retrieve gene relationships. These pair-wise relationships have no indication of the source pathway name. We need to map these relationships to their corresponding pathways. MouseCyc and BioCyc provide a file that clearly records all the genes in each pathway. Using this information, we are able to map the gene relationships to their corresponding pathways.

Converting the relationship of genes in complexes (groups) into binary gene pair relationships may not be the most ideal format for some users, who wish to refer to the original protein complexes information in pathways. For KEGG and WikiPathways we also maintain a "group-gene list" which specifically retains the original format of genes in the groups. The groups in this "group-gene list" are not integrated, as we have done for the pathway-gene and pathway-gene pair relationships, since maintaining this "group-gene list" is mainly to prevent information loss and to give users more precise original information that may not be easily reconstructed from the integrated pathway-gene pair relationships. We normalize the gene IDs in the "group-gene list" to IntPath unified gene IDs and store the list in a simple text file. Users can download this list along with other pathway-gene and pathway-gene pair relationships in the form of compressed text files from IntPath.

Normalization of gene names is done using gene ID mapping files downloaded from a variety of databases including NCBI [16], KEGG [1], UniProt [17], HGNC [18], MouseCyc [6], BioCyc [4,5], and BioMart [19]. The gene relationships from different databases are mapped to the IntPath unified relationships listed in Table 1.

### Evaluation of normalized pathway genes and gene pairs from different databases

After we have obtained the pathway-gene and pathway-gene pair relationships from different pathway databases, agreement among the databases can be analyzed. These agreement analyses are crucial for the downstream applications of IntPath. We examine the agreement among the different pathway databases in three aspects: (i) agreement of genes and gene pairs in different databases, (ii) agreement of the pathways in different databases, and (iii) agreement of genes and gene pairs of the same pathway in different databases.

**Figure 1:**
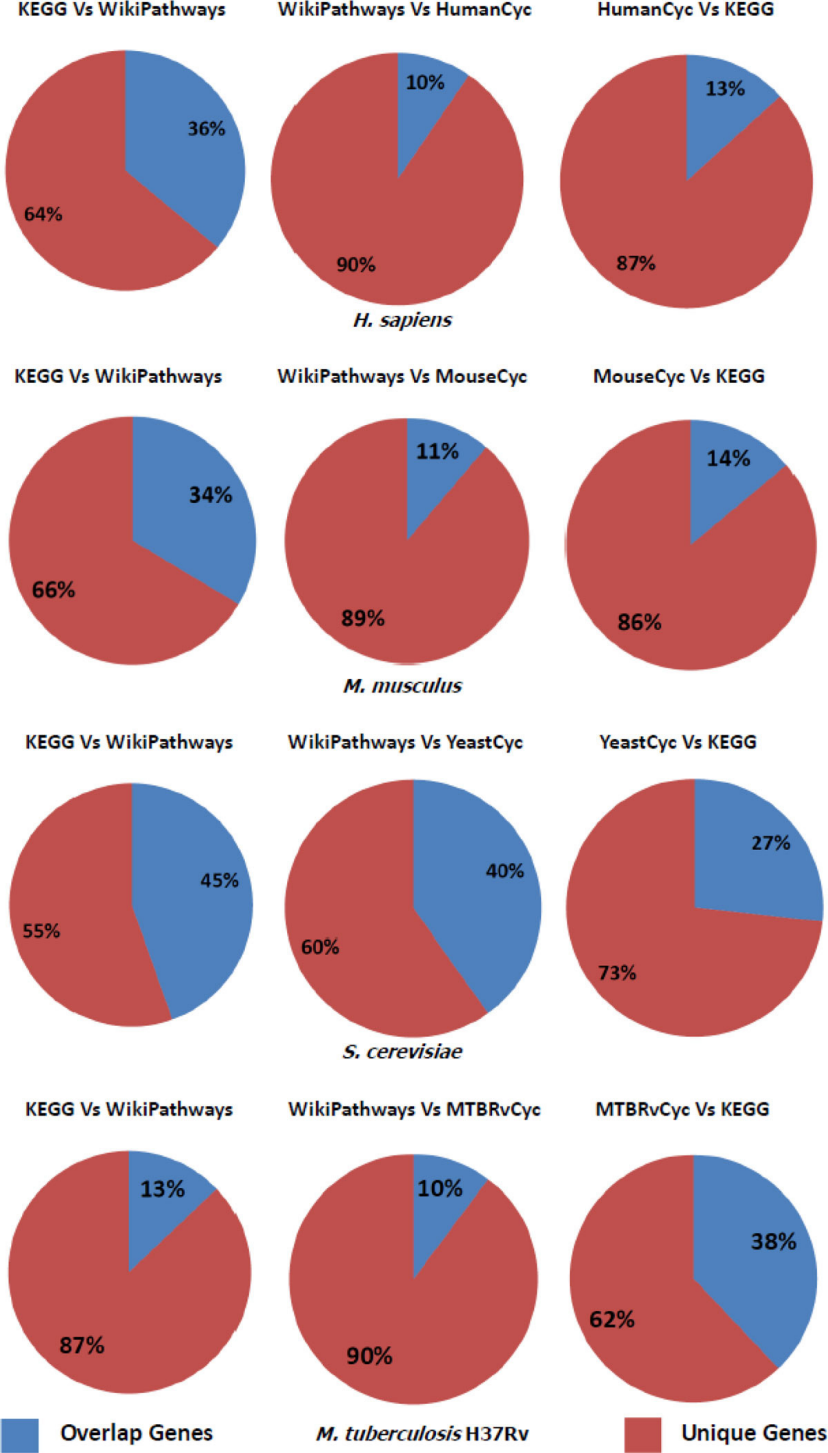
**Pie charts depicting overlapping gene proportions**. The red part refers to the proportions of unique genes while the blue part refers to proportions where there is an overlap of genes.

**Figure 2:**
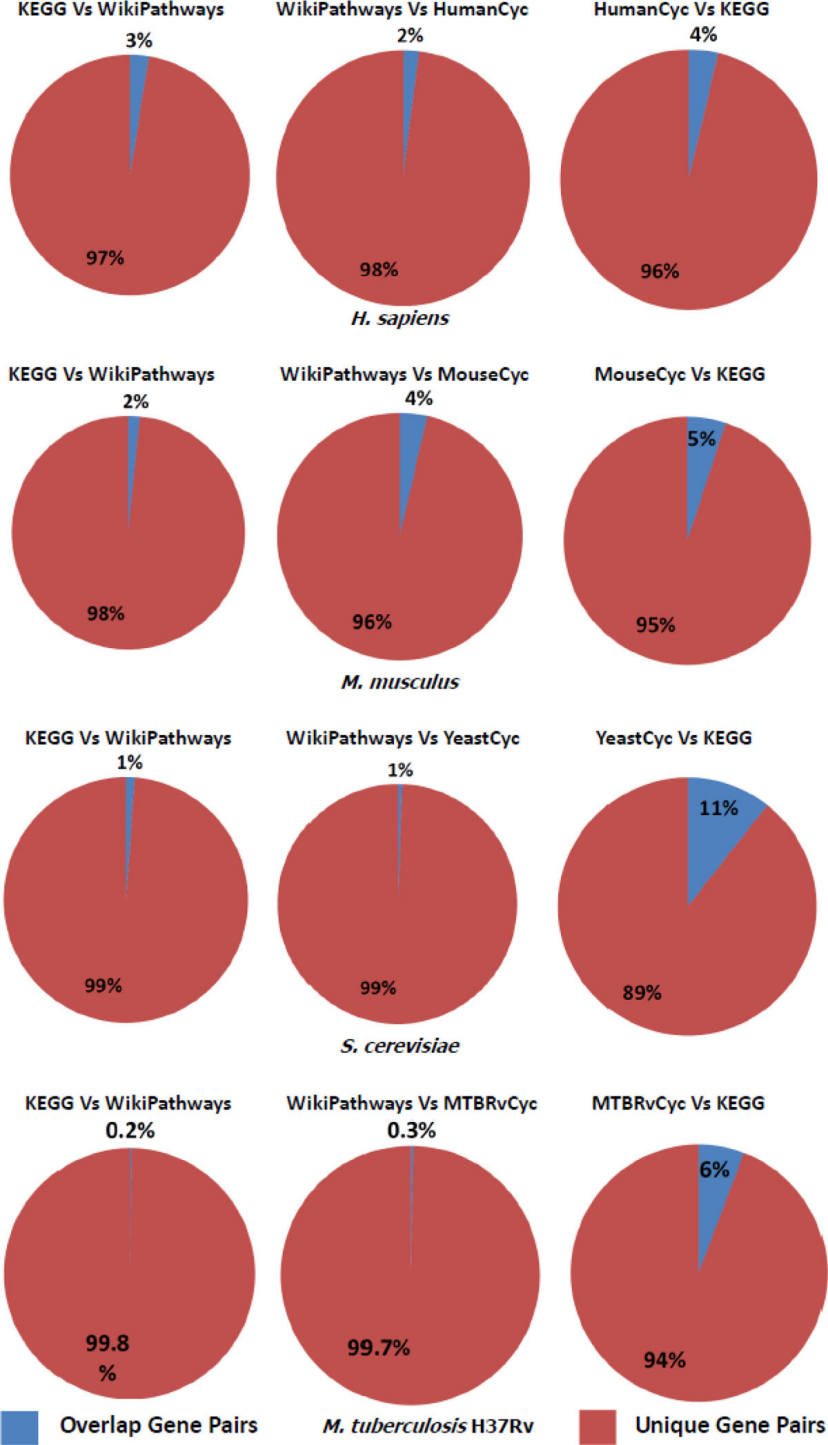
**Pie charts depicting overlapping gene pair proportions**. The red part refers to the proportions of unique gene pairs while the blue part refers to proportions where there is an overlap of gene pairs.

After normalization the statistics about pathway number, gene number and gene pair number in each of the source databases can be found in Table 2. To calculate the agreement of genes and gene pairs in different databases, we obtain all the non-redundant genes and gene pairs (without considering the types of relationships) in different databases. We then calculate how many genes and gene pairs are common between two databases being compared. The Jaccard coefficient between two datasets being compared is calculated. Results are shown in the form of pie charts in Figure 1 and 2, the detail statistics are listed in Table 3 and Table 4.

**Table 2:** The number of pathways, genes and gene pairs from different databases after normalization.

*H. sapiens*	KEGG	WikiPathways	HumanCyc
Pathways	237	135	290
Genes	5,935	3,445	1,082
Gene Pairs	29,566	18,035	5,961

*M. musculus*	KEGG	WikiPathways	MouseCyc

Pathways	218	140	323
Genes	6,306	4,084	1,194
Gene Pairs	32,235	25,004	10,792

*S. cerevisiae*	KEGG	WikiPathways	YeastCyc

Pathways	98	125	184
Genes	1,735	863	542
Gene Pairs	2,922	57	1,440

*M. tuberculosis *H37Rv	KEGG	WikiPathways	MTBRvCyc

Pathways	110	8	234
Genes	1,078	152	493
Gene Pairs	3,775	62	2,764

Summary of the number of pathways, genes, and gene pairs after normalization from different databases.

**Table 3:** Summary of overlapping gene proportions.

*H. sapiens*	KEGG vs WikiPathways	WikiPathways vs HumanCyc	HumanCyc vs KEGG
Overlap Genes	2,485	396	824
Unique Genes	4,410	3,735	5,369
Jaccard Coefficient	0.360	0.096	0.133

*M. musculus*	KEGG vs WikiPathways	WikiPathways vs MouseCyc	MouseCyc vs KEGG

Overlap Genes	2,611	532	919
Unique Genes	5,168	4,214	5,662
Jaccard Coefficient	0.336	0.112	0.140

*S. cerevisiae*	KEGG vs WikiPathways	WikiPathways vs YeastCyc	YeastCyc vs KEGG

Overlap Genes	801	402	480
Unique Genes	996	601	1,317
Jaccard Coefficient	0.446	0.400	0.267

*M. tuberculosis *H37Rv	KEGG vs WikiPathways	WikiPathways vs MTBRvCyc	MTBRvCyc vs KEGG

Overlap Genes	141	60	432
Unique Genes	948	525	707
Jaccard Coefficient	0.129	0.103	0.379

Summary of the number of overlap genes, number of unique genes, and Jaccard coefficient among three representative databases.

**Table 4:** Summary of overlapping gene pair proportions.

*H. sapiens*	KEGG vs WikiPathways	WikiPathways vs HumanCyc	HumanCyc vs KEGG
Overlap Gene Pairs	1198	468	1,270
Unique Gene Pairs	45,205	23,060	32,987
Jaccard Coefficient	0.026	0.020	0.037

*M. musculus*	KEGG vs WikiPathways	WikiPathways vs MouseCyc	MouseCyc vs KEGG

Overlap Gene Pairs	875	1,242	2,068
Unique Gene Pairs	55,489	33,312	38,891
Jaccard Coefficient	0.016	0.036	0.050

*S. cerevisiae *	KEGG vs WikiPathways	WikiPathways vs YeastCyc	YeastCyc vs KEGG

Overlap Gene Pairs	35	9	419
Unique Gene Pairs	2,909	1,479	3,524
Jaccard Coefficient	0.012	0.006	0.106

*M. tuberculosis *H37Rv	KEGG vs WikiPathways	WikiPathways vs MTBRvCyc	MTBRvCyc vs KEGG

Overlap Gene Pairs	9	8	358
Unique Gene Pairs	3,819	2,810	5,823
Jaccard Coefficient	0.002	0.003	0.058

Summary of the number of overlap gene pairs, number of unique gene pairs, and Jaccard coefficient among three representative databases.

**Figure 3:**
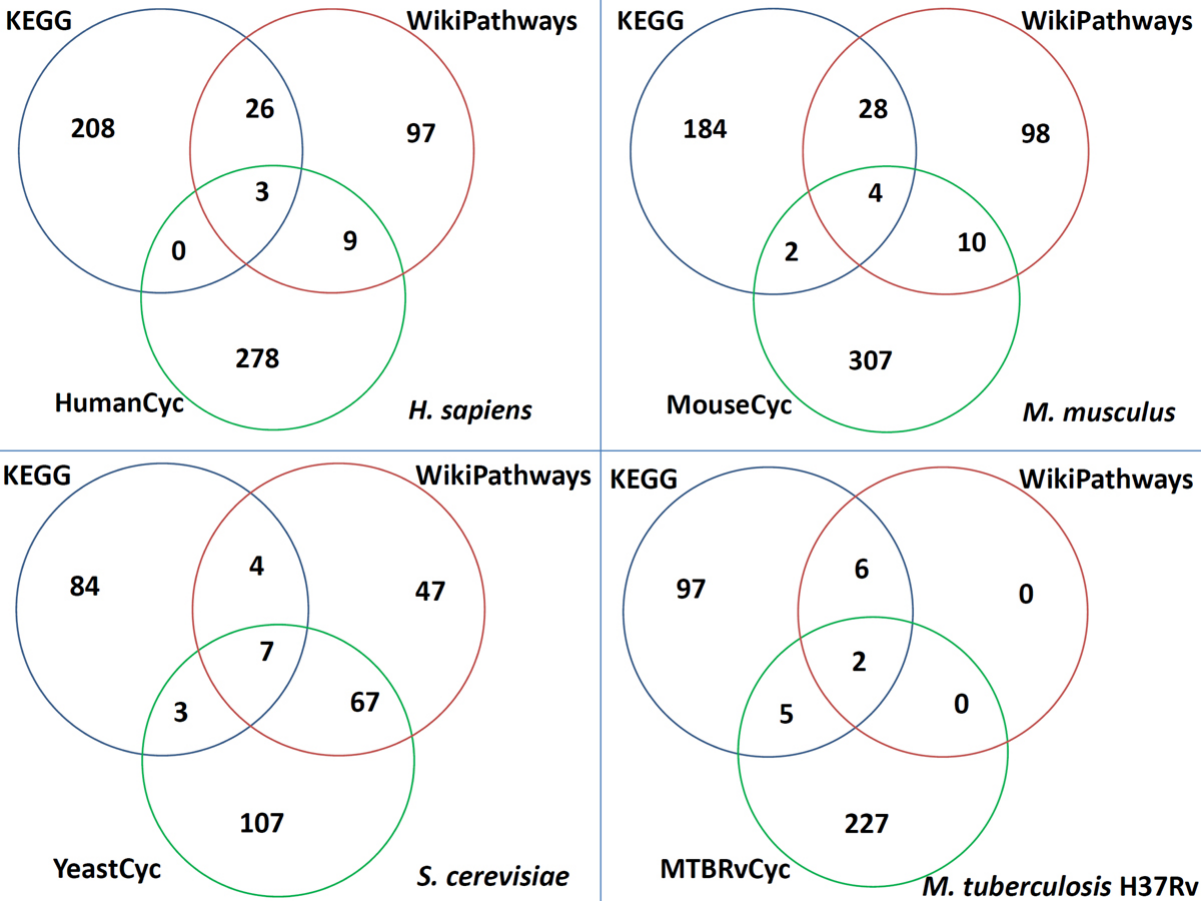
**Venn diagram of pathways in different databases**. Venn diagram depicting overlapping pathways across the three databases.

To analyze the agreement of the pathways in different databases, we only look at the pathway names in different databases, and calculate how many pathways two databases have in common. To find similar pathway names, we implement a "Longest Common Substring" algorithm. Our program can detect similar pathway names very accurately; detailed techniques will be explained in the following section. In this analysis we only search the related pathway between databases rather than within databases. The results are presented in Figure 3.

The two experiments above are analyses at the database level. Next, to analyze--at the pathway level--the agreement of genes and gene pairs of the same pathway in different databases, We calculate the overlap of the genes and gene pairs in the chosen pathway in different databases. The results are summarized in Table 5.

**Table 5:** Table showing data overlap for same chosen pathways in difference source databases.

*M. musculus*	TCA cycle pathway	KEGG vs WikiPathways	KEGG vs MouseCyc	MouseCyc vs WikiPathways
Gene	Count	31 vs 30	31 vs 13	13 vs 30
	Overlap	24	13	11
	Jaccard Coefficient	0.65	0.42	0.34
Gene Pair	Count	100 vs 30	100 vs 24	24 vs 30
	Overlap	10	9	7
	Jaccard Coefficient	0.083	0.078	0.149

*H. sapiens *	Fatty Acid Biosynthesis	KEGG vs WikiPathways	KEGG vs HumanCyc	HumanCyc vs WikiPathways

Gene	Count	6 vs 22	6 vs 2	2 vs 22
	Overlap	3	2	1
	Jaccard Coefficient	0.12	0.33	0.04
Gene Pair	Count	12 vs 29	12 vs 2	2 vs 29
	Overlap	1	1	0
	Jaccard Coefficient	0.025	0.077	0.0

*M. tuberculosis *H37Rv	TCA cycle pathway	KEGG vs WikiPathways	KEGG vs MTBRvCyc	MTBRvCyc vs WikiPathways

Gene	Count	35 vs 34	35 vs 10	10 vs 34
	Overlap	34	10	10
	Jaccard Coefficient	0.97	0.29	0.29
Gene Pair	Count	107 vs 37	107 vs 19	19 vs 37
	Overlap	3	9	5
	Jaccard Coefficient	0.021	0.077	0.098

This table shows the calculation of gene/gene pair differences and overlap between the different source databases for the same chosen pathways.

### Integration of pathway-gene and pathway-gene pair relationships

From the analyses above, we realize the lack of comprehensiveness and consistency of different pathway databases at both the database level and the pathway level. Hence, we should use the integrated information from all the databases rather than rely on any single source. The inconsistent referrals to pathway names further strengthen the necessity of integrating the pathway-gene and pathway-gene pair relationships from different databases into one unified and comprehensive information source.

To find all related pathways both among and within databases which have inconsistent referrals to pathway names (both name variations and different levels of emphases), we implement a refinement of the Longest Common Substring (LCS) algorithm to identify related pathway names. LCS was shown by [11] to be superior for identifying related pathways in different databases, compared to approaches based on large overlap of genes and interacting gene pairs.

The common LCS algorithm based on dynamic programming works like this: when comparing two strings, the more similar they are, the higher alignment score they have. In our program, the alignment score is the number of aligned characters. We also compute the alignment ratio, which is two times the alignment score divided by the sum of the length of the two strings. To identify the related pathway names in two databases' pathway name lists (*x *and *y*), we iterate each name in the list *x *and search against all the names in the list *y*; for each name in *x*, we report the best hit in *y*. "Best hit" means that, for each name from *x*, when searched against all the names in *y*, the one that gets the highest alignment ratio is reported as the best hit for this round. We do not use the alignment score to report best hits because the alignment ratio proves to perform better. This is because some related pathway names do not have very high alignment scores due to the short length of two strings, but the similarities of the two strings can be revealed accurately by the high alignment ratio when compared to other not-so-similar long strings in a single round of search. For example, suppose the name *Xa *is very short and is searched against the name list *y*. Suppose there is a very similar short name *Y a *which aligns all characters in *Xa *except one character. Suppose there is also a very different but long name *Y b *which aligns all characters in *Xa*. It is obvious that the alignment score *Xa *- *Ya *is lower than the alignment score *Xa *- *Yb*, while the alignment ratio of *Xa *- *Ya *is the higher of the two. Thus using the alignment score to report the best hit is not as good as using the alignment ratio.

From many background experiments, we realize that relying only on best hits can result in some noise, since many pathway names in the list *x *may not have any related pathway names in the list *y*. Our strategy is to introduce more stringent requirement to increase the precision of the reported best hits. We require that either of the following two additional (empirically determined) conditions to be satisfied:

1. Alignment score *>*the length of shorter string - 1 & alignment ratio >= 0.5, or

2. Alignment ratio *>*0.91

Combined with this additional requirement, our program achieves high precision and recall in identifying related pathway names. Nevertheless, a small number of pathways which do not describe the same pathway, but have very similar names, are still incorrectly identified by the methods described above as related pathways. "VEGF signaling pathway" and "EGFR1 Signaling Pathway", "T Cell Receptor Signaling Pathway" and "B Cell Receptor Signaling Pathway", etc. are examples of this kind of mismatches. Our approach to solve this problem is by using a "error-prone words pair list" to filter potential mismatches. For example, if in a candidate related pathway pair, one pathway name has one partner of an "error-prone words pair"(EGFR1) and the other pathway name contains the other partner in the "error-prone words pair"(VEGF), this pair of candidate related pathways is discarded by our program. This approach successfully gets rid of mismatched pathways without compromising the identification of related pathways. Although a little manual curation is needed for initializing the "error-prone words pair list", the curation work load is much less after the first time, since only a few changes or supplementations of "error-prone words pair list" are needed when processing different groups pathway names. Moreover, it is suitable for many different pathways in different organisms.

We run our program to compare pathway names within each database and between the databases. After obtaining all the related pathways, our program uses a disjoint set data structure to store all the identified related pathways and then groups together all the related pathways under a general pathway name. The general pathway name is chosen as the shortest pathway names from among the identified related pathways. The shortest pathway name is usually suitable to be the name of the integrated pathway. However, in some cases, the shortest name contains "suffix" or "prefix"--like "I", "II"--that causes the integrated pathway name to give the wrong idea of describing only a specific aspect of the integrated pathway. So our program removes such suffixes and prefixes when generating integrated pathway names. In addition, there are also a small number of cases where several similar pathways are included in one pathway name--an example is shown in the last row of Table 6. In these cases, the shortest name is not appropriate as the name of the integrated pathway. For these small number of cases, we replace the keyword of the integrated pathway name to cover more pathway information. After all the processing steps described above, we can be sure that the integrated pathway names in IntPath is correct and accurate. The numbers of identified related pathway names are listed in Table 7. The number of pathways, average number of genes per pathway, and average number of gene pairs per pathway in each database, before and after this integration, is given in Table 8.

**Table 6:** Examples of inconsistent referrals to pathway names in *M. musculus*.

IntPath	KEGG	WikiPathways	MouseCyc
Fatty Acid	Fatty acid	Fatty Acid	1. fatty acid biosynthesis initiation II
Biosynthesis	biosynthesis	Biosynthesis	2. very long chain fatty acid biosynthesis
			3. fatty acid biosynthesis initiation III
Cholesterol		Cholesterol	1. cholesterol biosynthesis III (via desmosterol)
Biosynthesis		Biosynthesis	2. cholesterol biosynthesis II (via 24,25-dihydrolanosterol)
			3. cholesterol biosynthesis I
			4. superpathway of cholesterol biosynthesis
TCA cycle	Citrate cycle (TCA cycle)	TCA cycle	TCA Cycle
Glycolysis and Gluconeogenesis	Glycolysis/Gluconeogenesis	Glycolysis and Gluconeogenesis	1. glycolysis I 2. glycolysis II

The table shows several examples of the same pathways with inconsistent referrals to pathway names in different databases.

**Table 7:** Number of related pathways.

*H. sapiens*	KEGG	HumanCyc	WikiPathways
KEGG	5	3	29
HumanCyc	3	34	12
WikiPathways	29	12	4

*M. musculus*	KEGG	MouseCyc	WikiPathways

KEGG	6	6	32
MouseCyc	6	61	14
WikiPathways	32	14	10

*S. cerevisiae *	KEGG	YeastCyc	WikiPathways

KEGG	1	10	11
YeastCyc	10	25	74
WikiPathways	11	74	15

*M. tuberculosis *H37Rv	KEGG	MTBRvCyc	WikiPathways

KEGG	1	7	8
MTBRvCyc	7	35	2
WikiPathways	8	2	0

Summary of the number of identified related pathways within and among databases.

**Table 8:** Summary of number of pathways, average number of genes per pathway and average number of gene pairs per pathway before and after integration.

*H. sapiens*	No. of Pathways BEFORE integration	Average No. of genes/pathway	Average No. of gene pairs/pathway
WikiPathways	135 pathways	46.3	166.2
HumanCyc	290 pathways	7.2	33.0
KEGG	237 pathways	72.4	171.3

*H. sapiens *	No. of unique Pathways AFTER integration	Average No. of genes/pathway	Average No. of gene pairs/pathway

WikiPathways	100 pathways	42.7	157.4
HumanCyc	225 pathways	7.2	31.6
KEGG	201 pathways	72.6	165.3
Integrated Pathways	57 pathways	59.5	263.6

*M. musculus *	No. of Pathways BEFORE integration	Average No. of genes/pathway	Average No. of gene pairs/pathway

WikiPathways	140 pathways	57.8	209.1
MouseCyc	323 pathways	8.0	61.4
KEGG	218 pathways	74.6	194.8

*M. musculus *	No. of unique Pathways AFTER integration	Average No. of genes/pathway	Average No. of gene pairs/pathway

WikiPathways	97 pathways	56.8	242.8
MouseCyc	204 pathways	7.4	43.0
KEGG	172 pathways	77.9	187.3
Integrated Pathways	85 pathways	52.6	260.9

*S. cerevisiae *	No. of Pathways BEFORE integration	Average No. of genes/pathway	Average No. of gene pairs/pathway

WikiPathways	125 pathways	11.8	0.5
YeastCyc	184 pathways	6.5	13.4
KEGG	98 pathways	35.2	34.7

*S. cerevisiae *	No. of unique Pathways AFTER integration	Average No. of genes/pathway	Average No. of gene pairs/pathway

WikiPathways	45 pathways	15.1	0.2
YeastCyc	85 pathways	5.8	11.6
KEGG	80 pathways	38.0	35.0
Integrated Pathways	76 pathways	14.1	25.2

*M. tuberculosis *H37Rv	No. of Pathways BEFORE integration	Average No. of genes/pathway	Average No. of gene pairs/pathway

WikiPathways	8 pathways	22.3	7.8
MTBRvCyc	234 pathways	5.7	18.9
KEGG	110 pathways	32.5	47.5

*M. tuberculosis *H37Rv	No. of unique Pathways AFTER integration	Average No. of genes/pathway	Average No. of gene pairs/pathway

WikiPathways	0 pathways		
MTBRvCyc	171 pathways	5.9	21.0
KEGG	94 pathways	35.4	51.7
Integrated Pathways	35 pathways	12.3	25.4

The table below shows the number of pathways from major pathway databases before and after integration.

### IntPath web interface and web service

IntPath is developed using JSP and MySQL. The web service is created and published using AXIS2.

## Results

### Extraction and normalization of pathway-gene and pathway-gene pair relationships

In order to overcome the limitation of incompatible data formats, we directly extract from the XML files (KGML, GPLM, BioPAX) of each pathway database and obtain the gene relationships. To deal with inconsistent molecular representations, we normalize the gene representations into a unified gene ID. The IntPath unified gene ID (which adopts a set of the most commonly used gene names) is compatible with the gene names used in most public repositories. A summary of the number of pathways, genes and gene pairs from different databases after normalization is given in Table 2. To tackle inconsistent molecular relationship representations, we also normalized the relationships of different databases into the IntPath unified relationship types as shown in Table 1.

### Evaluation of normalized pathway genes and gene pairs from different databases

After obtaining the normalized pathway-gene and pathway-gene pair relationships, we are able to analyze the comprehensiveness and agreement among the different pathway databases on different aspects.

The results from analyzing the overlap of genes and gene pairs in different databases are presented in pie charts in Figures 1 and 2. The detailed statistics are summarized in Tables 3 and 4. These results prove that the overlap of genes and gene pairs in different databases are very low. This result is in accord with similar experiments done on human pathway databases [11].

From the results on the overlap of the pathways in different databases we can see there is also a strikingly low overlap of pathways among the different databases; see Figures 3. This demonstrates the obvious low level of comprehensiveness in the databases analyzed, also in accord with the experiments on human pathway databases described in [11].

Zooming in from the database level to the individual pathway level, we analyze the agreement of genes and gene pairs of the same pathway in different databases. The results are listed in Table 5. The agreement of different databases at the pathway level is also not as high as we expected (especially for gene pairs), which proves the low level of consistency between these databases on the same pathway.

The comparative analyses from the above three aspects clearly exhibit the incomprehensiveness and inconsistency among the pathway databases. This suggests that the integration of the extracted and normalized information from different databases into a unified and comprehensive resource is very necessary.

### Integration of pathway-gene and pathway-gene pair relationships

The results above demonstrate that relying only on a single source of pathway information from any of the databases is not reasonable. Moreover, we have also discovered the problem of inconsistent referrals to pathway names. Table 6 lists some examples of the same pathway under inconsistent names in different databases. Those are just a few typical examples; there are many pathways with similar situations which need to be properly addressed. Therefore, it is of great necessity to integrate all the pathway-gene and pathway-gene pair relationships from different databases into a comprehensive and unified source.

In the integrated pathways, all the related pathways with inconsistent names should be merged. (i) The inconsistent referrals to pathway names are partially caused by the different levels of emphases on the same pathway in different databases. One database (BioCyc) may emphasize on some very specific aspects of a certain large pathway; so this large pathway is broken up in this database into different pathways with similar/related names, yet all describing the detailed aspects of the original large pathway; see Table 6. However, the other two databases may emphasize on a more general level and, therefore only use a general and often shorter pathway name. When merging pathways from different databases into integrated pathways, we should unify the different levels of emphases. We decide to choose a more general level rather than a detailed level. (ii) When merging the same pathways with different levels of emphases in different databases, if we have already merged one detailed-level pathway into a general-level pathway, all other related detailed-level pathways in the databases should be merged into this general-level pathway. After merging all the related pathways we should use a general pathway name (usually the shortest one) to represent the integrated pathway. (iii) The distinct differences between our integrated pathway gene relationships and conventional pictorial pathway map indicate a more general level is suitable. We are primarily focusing on gene relationships, but not on other the relationships in the pathways (protein-compound relationships, compound-compound relationships, and so on.) in this version of IntPath. This emphasis results in less enthusiasm on the detailed level of individual pathways, and we lack sufficient information (just gene relationships) to emphasize on the detailed level in most cases. (iv) The common problem of gene relationships is the sparseness in each pathway; and putting emphasis on the detailed aspect of certain pathway could render the data in a single pathway too sparse to be useful.

For the reasons listed above, we should merge all the related pathways under the same general name into one comprehensive pathway (among and within databases). And after merging, we should use the general pathway name which is usually the shortest name among all the comparing pathway names.

The results of identified related pathways both within and among databases are summarized in Table 7. From the number of related pathways within databases, we find BioCyc and MouseCyc emphasize more on the detailed aspect of pathways; therefore, more related pathway names are identified. In IntPath, all the related pathways within and among databases are grouped together with the integrated pathway name. The number of pathways, average number of genes per pathway and average number of gene pairs per pathway, before and after integration, in the four IntPath included organisms are given in Table 8. The statistics listed in Table 8 clearly show that in integrated pathways there is a significant increase of average node degree (average node degree = average no. of gene pairs per pathway/average no. of genes per pathway), which means significant increase of gene relationships of each gene on average in the integrated pathways. There is also a considerable increase of average no. of gene pairs per pathway in the integrated pathways, which indicates richer gene relationships on average in each pathway. In some sense, the integration approach partially solves the sparseness of pathway-gene relationships in MouseCyc and BioCyc.

We accomplished in IntPath the integration of pathway-gene and pathway-gene pair relationships, achieving compatible data formats, consistent molecular representations, consistent relationship representations, consistent referrals to pathway names and comprehensive data.

### IntPath web interface and web service

**Figure 4:**
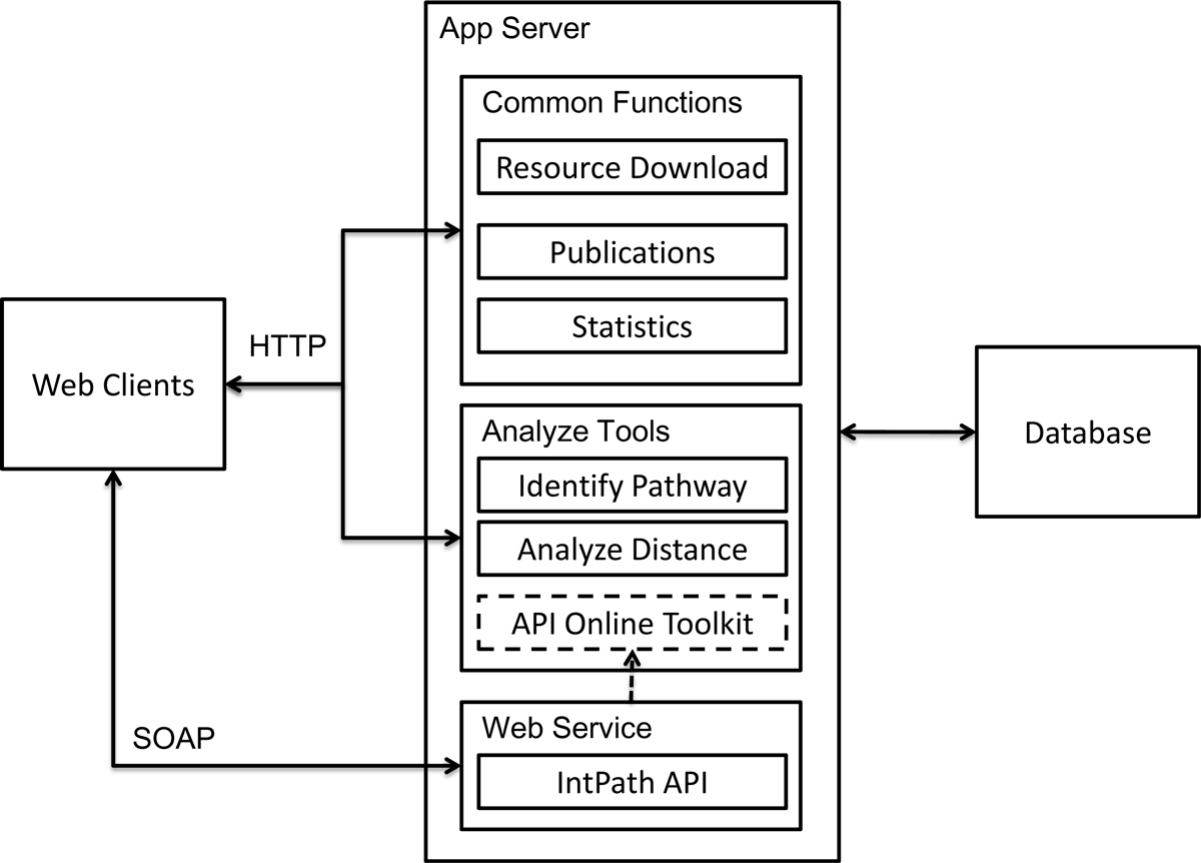
**IntPath system overview**. This figure shows the components of IntPath database, the relationships between those components and a clear indication on which components are supported by web service and which are supported by web interface.

**Figure 5:**
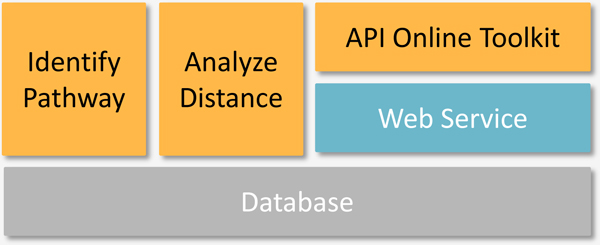
**Core functions of IntPath**. This figure shows the core functions of IntPath, the relationships between those core functions, database and web service.

The web interface of IntPath comprises the following parts: Home, Gene List Analysis Tools (Identify Pathways and Analyze Distances), API Toolkit, Statistics, Tutorial, and Download. In order to facilitate convenient access of IntPath data through local programs, the API functions are also supported by IntPath web service. An overview of the IntPath system is shown in Figure 4. The core functions of IntPath are represented in Figure 5. An explanation of each part is given below.

Home: It is to introduce the objective of IntPath, what the major contribution of this database is and what the specific problems that we wish to solve through this database are. We also indicate the analysis tools supported in this database, the publications related to these analysis tools, and which species are currently included in our database. This Home page of IntPath is a summary of the general information of the database.

Identify Pathways: The function of "Identify Pathways" uses the hyper-geometric test to find the most significant pathways given an input gene list. Through this tool, users can have a clear insight of which pathway is most related to the input gene list. For each result returned, details like p-value are also given.

Analyze Distances: The function of "Analyze Distances" is to tell the similarities between the two input gene lists from a pathway perspective. To perform the distance analysis, first the hyper-geometric test is used to find the most significant pathways of the two input gene lists, then the Floyd-Warshall algorithm is used to calculate the "distances" between the two pathways. STRING PPI datasets (version 9.0) is used in the distance calculation between two pathways in the current version of IntPath (V2.0). The "distances" provide a reference in telling the relationships between two specific pathways, and it can be very useful, e.g., in identifying how "far" it will take for a normal pathway to transform into a diseased pathway. For a detailed explanation of "Analyze Distances" and its application in biomedical research, please refer to methods described in [20].

Statistics: This statistics section gives users an overall insight of IntPath. Users can easily get the following statistics: number of genes, number of gene pairs, number of integrated pathways, number of original KEGG pathways, number of original WikiPathways pathways, number of original BioCyc(MouseCyc) pathways, and number of source databases. The default option is "All statistics" which displays all the statistics listed above.

API Toolkit: We provide powerful as well as flexible API functions of our IntPath database. Users can both call the API functions using their local programs through IntPath's web service or using API functions by directly retrieving information through IntPath's web interface. The following API functions are supported, getGeneID, getDBPathways, getPathway, getPathwayGenes, getGenePathways, getPathway-Interactions, getPathwayDifference, getIntPathGenes, getIntPathGenePairs and getIntPathPathways. The explanation and user guide of each API function can be found in the Tutorial page.

Download: Some users may have other requirements of data analyses that are not met by IntPath in the current version. Some users may also have different application purposes of IntPath. To cope with a variety of needs, we release all our IntPath data in this "Download" section, where users can obtain all IntPath data in two different formats: (1) text format (*.txt), this compressed package includes three text files, (a) the integrated pathway-gene relationships, (b) the integrated pathway-gene pair relationships and (c) the normalized group-genes list; and (2) sqldump format (*.sql), which is based on the integrated data we have prepared and stored in 6 tables in each sqldump (each organism is a separate sqldump).

## Discussion

### Comments on WikiPathways

The "wiki-style" of WikiPathways makes this database more casual than other databases. It is good for the community to freely maintain and share knowledge through WikiPathways. On the other hand, it causes many problems for automatic information retrieval. One of the limitations is the slight inconsistency among the formats of GPML as mentioned before--some key tags can be upper or lower cases. GPML is more different from other XML formats. GPML emphasizes more on pictorial information; therefore, most of the objects on the file are more likely to be recorded for their positional information. Worse, some GPML files even do not have a "graphID" record; and for these GPML files, whole information of certain pathways is given by the positional information on the pathway map. For these GPML files, judging the relationships between two genes is solely dependent on the positional information. It may be easy for the human eye to look at the pictorial format of the pathway map; but it is hard for computer programs to retrieve accurate information automatically. Attempts on spatial clustering have been made. But these attempts also introduce a substantial amount of noise. Therefore we decide to discard this noisy information at the current stage.

Recently, WikiPathways begins to support web service and BioPAX. We have tried solving the problem mentioned above using WikiPathways web service and directly extracting from BioPAX format; but no improvement has been achieved.

Web service has not solved the problem of those GPML files that do not have "graphID" record. For example, our program fails to extract reliable gene relationships from the pathway "Mm Androgen Receptor Signaling Pathway WP252_35669" by calling the WikiPathways API function "find Interactions". It is supposed to find interactions defined in WikiPathways' pathways. In our experiments, it works in finding interactions in other pathways. Extensive experiments have been made using different ways to call the "find-Interactions" function. Yet nothing related to the WP252 pathway is returned. On the png graph we can see there are lots of interactions in this WP252 pathway. These kinds of experiments have been attempted many times on several pathways. All have failed to find the "interactions" or gene relationships in the specific pathways that lack "graphID" entry.

We turn to BioPAX files which have recently been supported on WikiPathways for a solution. We specifically run our program on the pathway BioPAX files whose corresponding GPML files do not have "graphID" records. Our program successfully retrieved gene relationships from BioPAX files in BioCyc and MouseCyc, but not on these specific BioPAX files in WikiPathways (for example, "Sc Cell Cycle and Cell Division WP414_21554" and "Sc Glycolysis and Gluconeogenesis WP515_42806"). We also try to visualize those specific BioPAX files on Cytoscape; but no relationship can be visualized from these files.

The gene ID problems in WikiPathways is also quite serious. There are two places to retrieve gene ID information from the GPML "</DataNode>" entry, one is from "TextLabel" and the other is from "<Xref Database"IDs. Usually gene IDs in "TextLabel" are gene symbol, while gene IDs in "<Xref Database" can be the gene IDs from different public databases, like Entrez, Ensembl, UniProt, and so on. Getting gene ID information from both of these two fields is necessary. It is not uncommon for the WikiPathways database to have errors and problems in both fields. In most cases, erroneous gene IDs from "TextLabel" also do not have any information in "<Xref Database". The erroneous gene IDs can be gene symbols or EC numbers that cannot be found in the target organism to which the pathway map belongs; they can also be common gene names without any information in "<Xref Database", or they can be just upper- or lower-case flaws. In our program, both information from the two fields,"TextLabel" and "<Xref Database" are retrieved. For gene IDs where information from both of these fields are problematic, manual curation is adopted to deal with them, generally by removing them from IntPath.

### Access, update and extension of IntPath

IntPath and all its data have been released online at http://compbio.ddns.comp.nus.edu.sg:8080/IntPath. As some studies are already using data in IntPath [21], we believe our work here can facilitate a variety of works that need to refer to pathway information.

IntPath heavily depends on source pathway data from all the pathway databases and most databases update quite frequently. The important question is: Can we keep our data updated in a timely fashion? The answer is: Yes.

The "IntPath Data Preparation" program is streamlined and automated in performing the extraction, normalization, integration processes and directly outputing into MySQL databases and text files. For organism already included in IntPath, running the program for each update takes a short time; and we will maintain a regular update of IntPath in the long term. Another key question is whether we can extend our approach to other organisms. Currently, we have already included four organisms--*S. cerevisiae*, *M. tuberculosis *H37Rv, *H. Sapiens *and *M. musculus*--and we will include more in future releases of IntPath. Extending the methodology to include other organisms just needs modifying the regular expressions for extracting GPML and KGML files; preparing the gene ID mapping files; manually correcting some possible errors of the gene IDs introduced by the source databases (like WikiPathways gene ID problems) and, if necessary, updating the "error-prone words pair list"; and reviewing integrated pathway names. Therefore, the whole process of including other organisms in IntPath takes a short time. We will include more model organisms and important pathogens in IntPath in future releases.

### Outlook of IntPath

In the near future, more functions and analysis tools will be supported in IntPath--for example, clustering algorithms for microarray studies using the IntPath data as background knowledge, visualization tools of interaction and relationship, more powerful algorithms to identify pathways given user-specified input gene lists, and more API functions. Moreover, in this version of IntPath we only take gene relationships into account; in a future version, IntPath will also consider other important relationships in the pathways--like protein-compound relationships, compound-compound relationships, and so on. Meanwhile, in future releases, more organisms will be included. We wish our continuing effort can make IntPath one of the most useful databases in pathway studies that can benefit a variety of related researches.

## Conclusion

The five limitations of current pathway databases that hamper effective use of pathway information have been overcome in this work. We solve the problem of incompatible data formats in different databases by extracting the pathway-gene and pathway-gene pair relationships. The limitations of inconsistent molecular representations and inconsistent molecular relationship representations have been overcome by our normalization of the data into common gene name representations and common relationship types which are compatible with other database. The problems of inconsistent referrals to pathway names and incomprehensive data from different databases have been solved by the integration of pathway-gene and pathway-gene pair relationships into a unified and comprehensive data source.

We achieve compatible data formats, consistent molecular representations, consistent relationship representations, consistent referrals to pathway names and comprehensive data in our IntPath database for several organisms--viz., *H. sapiens*, *S. cerevisiae*, *M. musculus *and *M. tuberculosis *H37Rv. IntPath can maintain a regular update in these organisms and, the methodology we describe here can be applied to other organisms straightforwardly.

We believe IntPath will not only facilitate convenient access of the integrated pathway gene relationship data for model organisms and important pathogens but also greatly boost data analysis and application to many related studies through the analysis tools and API functions provided in the database.

## Competing interests

The authors declare that they have no competing interests.

## Authors' contributions

This work was jointly conceived, planned, and written up by Limsoon Wong and Hufeng Zhou. The analytical experiments were performed by Hufeng Zhou. The IntPath data were prepared by Hufeng Zhou. The IntPath database web interface was designed and implemented jointly by Hufeng Zhou, Jingjing Jin, Bo Yi, Haojun Zhang as follows. Hufeng Zhou prepared the relational databases of all the included organism's data in MySQL. Jingjing Jin provided the draft codes of Analyze Distances, Identify Pathways and parts of API functions of IntPath web interface supporting PathwayAPI data. Hufeng Zhou improved and rewrote the draft codes provided by Jingjing Jin, except the codes of the Floyd-Warshall algorithm. Hufeng Zhou implemented the rest parts of the IntPath web interface. Hufeng Zhou and Haojun Zhang identified the seralizable-file bug of the draft codes and, together improved the web interface response speed of IntPath API online Toolkit. Hufeng Zhou prepared and collected protein-protein interaction data and the serializeable files for all the included organisms. Hufeng Zhou and Bo Yi developed the IntPath web service. Hufeng Zhou and Michal Wozniak configured the draft codes for Linux and identified missing packages and bugs of the draft codes.
